# Effectiveness of Contact Tracing for Viral Disease Mitigation and Suppression: Evidence-Based Review

**DOI:** 10.2196/32468

**Published:** 2021-10-06

**Authors:** Kelly Jean Thomas Craig, Rubina Rizvi, Van C Willis, William J Kassler, Gretchen Purcell Jackson

**Affiliations:** 1 Center for AI, Research, and Evaluation IBM Watson Health IBM Corporation Cambridge, MA United States; 2 Palantir Technologies Denver, CO United States; 3 Vanderbilt University Medical Center Nashville, TN United States

**Keywords:** contact tracing, non-pharmaceutical interventions, pandemic, epidemic, viral disease, COVID-19, isolation, testing, surveillance, monitoring, review, intervention, effectiveness, mitigation, transmission, spread, protection, outcome

## Abstract

**Background:**

Contact tracing in association with quarantine and isolation is an important public health tool to control outbreaks of infectious diseases. This strategy has been widely implemented during the current COVID-19 pandemic. The effectiveness of this nonpharmaceutical intervention is largely dependent on social interactions within the population and its combination with other interventions. Given the high transmissibility of SARS-CoV-2, short serial intervals, and asymptomatic transmission patterns, the effectiveness of contact tracing for this novel viral agent is largely unknown.

**Objective:**

This study aims to identify and synthesize evidence regarding the effectiveness of contact tracing on infectious viral disease outcomes based on prior scientific literature.

**Methods:**

An evidence-based review was conducted to identify studies from the PubMed database, including preprint medRxiv server content, related to the effectiveness of contact tracing in viral outbreaks. The search dates were from database inception to July 24, 2020. Outcomes of interest included measures of incidence, transmission, hospitalization, and mortality.

**Results:**

Out of 159 unique records retrieved, 45 (28.3%) records were reviewed at the full-text level, and 24 (15.1%) records met all inclusion criteria. The studies included utilized mathematical modeling (n=14), observational (n=8), and systematic review (n=2) approaches. Only 2 studies considered digital contact tracing. Contact tracing was mostly evaluated in combination with other nonpharmaceutical interventions and/or pharmaceutical interventions. Although some degree of effectiveness in decreasing viral disease incidence, transmission, and resulting hospitalizations and mortality was observed, these results were highly dependent on epidemic severity (R0 value), number of contacts traced (including presymptomatic and asymptomatic cases), timeliness, duration, and compliance with combined interventions (eg, isolation, quarantine, and treatment). Contact tracing effectiveness was particularly limited by logistical challenges associated with increased outbreak size and speed of infection spread.

**Conclusions:**

Timely deployment of contact tracing strategically layered with other nonpharmaceutical interventions could be an effective public health tool for mitigating and suppressing infectious outbreaks by decreasing viral disease incidence, transmission, and resulting hospitalizations and mortality.

## Introduction

Contact tracing has a long history as an effective tool against infectious disease outbreaks, such as severe acute respiratory syndrome (SARS), Ebola, and monkeypox [1-3]. To mitigate the spread of disease, contact tracing involves interviewing people who are infected to identify which other individuals they might have exposed to the virus, finding those exposed contacts, isolating contacts who are infected, and placing exposed contacts in quarantine until they are not deemed infectious [4]. Public health agencies use contact tracing as one strategy among many to break the chain of viral transmission. As the number of vaccinated individuals increases and vaccine hesitancy and access issues persist, contact tracing remains a key strategy in the COVID-19 response to enable surveillance of the evolving COVID-19 pandemic [5].

Efforts to identify and support the contacts of those who have tested positive for COVID-19 and thus pose a risk for infecting others can be both resource- and labor intensive. Approaches to contact tracing have traditionally used telephone and in-person communication; however, newer approaches examine the use of mobile apps and leveraging data to track and trace social connections and potential exposures. Countries such as South Korea and Taiwan have touted the success of technology enablement; however, to date, evidence demonstrating a causal relationship between technology and COVID-19 mitigation is lacking [6-10]. The COVID-19 pandemic continues to overwhelm public health capacity due to the sheer numbers of those infected. Moreover, the pandemic is particularly challenging because of the large number of asymptomatic infections [11]. As such, the private sector will play a role in augmenting the public health response. Universities and businesses can collaborate with government agencies to facilitate contact tracing, and the use of technology can be an important enabler in this direction but concerns regarding privacy and effectiveness remain.

Like other nonpharmaceutical interventions (NPIs), the effectiveness of contact tracing is difficult to measure in real time owing to the lack of direct access to outcomes data and the reliance on surrogate data. Epidemiologists will determine the impact on COVID-19 with time, but in the interim, existing retrospective studies on the effectiveness of contact tracing to mitigate and suppress viral diseases offer a learning opportunity and valuable information to improve preparedness and response.

The objective of this study is to identify and synthesize evidence regarding the effectiveness of contact tracing on infectious viral disease outcomes. This evidence-based review focuses on studies describing the implementation and assessment of all forms of contact tracing with other NPIs and pharmaceutical interventions (PIs) by using single or multiple interventions during viral epidemics or pandemics.

## Methods

An evidence-based review was conducted using systematic methodology to identify literature from the PubMed database, including preprint medRxiv server content, related to the effectiveness of all forms and combinations of contact tracing approaches in viral epidemics or pandemics, including the COVID-19 pandemic. The search query (Table 1) included terms for contact tracing (eg, *contact tracing*, *case finding*, *case detection*) AND COVID-19, as well as other viral pandemics or epidemics (eg, *COVID-19*, *SARS-COV-2*, *2019-nCOV*, *severe acute respiratory syndrome coronavirus 2*, *novel coronavirus*, *influenza, flu*, *viral pandemic*, *viral epidemic*) AND effectiveness (eg, effective*, *efficacy*). The search dates were from database inception to July 24, 2020.

Outcomes of interest were measures of incidence, transmission, hospitalization, and mortality. Modeling studies with generalized statements of effectiveness were also included despite the lack of quantitative data. Primary and secondary articles were obtained; however, secondary articles were excluded with the exception of modeling studies and systematic reviews with inclusion criteria identical to this study. Single reviewer (KJTC) screening was conducted using a priori inclusion and exclusion criteria (Table 2). Data abstraction was completed from primary sources by one reviewer (KJTC), and quality control was undertaken by a second reviewer (RR, VCW) by using standardized forms. The study quality was assessed using Oxford Levels of Evidence [12] by a dual review (KJTC, RR, or VCW).

**Table 1:** Strategy used for the search conducted in MEDLINE (via PubMed).

Search number	Facet	Search terms	Search results (July 24, 2020)
1	Identify articles on NPIs^a^ of contact tracing used alone or in combination with other NPIs	(contact tracing[title] OR case finding[title] OR identify contacts[title]or detect case*[title] OR early detection[title] OR “contact tracing” [MeSH][title]) OR (non-pharmaceutical intervention* AND contact tracing)	17,896
2	Identify articles on viral epidemics or pandemics with a focus on COVID-19	*covid19[tiab] OR covid-19[tiab] OR* *severe acute respiratory syndrome coronavirus 2 or SARS-COV-2 OR 2019-nCoV OR novel coronavirus OR “COVID-19”[Supplementary Concept] OR viral epidemic[tiab] OR viral pandemic[tiab] OR influenza[tiab] or flu[tiab]*	140,427
3	Identify effectiveness outcomes	effective* OR efficacy OR effectiveness	8,348,810
4	Identify contact tracing effectiveness studies in viral epidemics or pandemics	#1 AND #2 AND #3	122

^a^NPI: nonpharmaceutical intervention.

**Table 2:** *A priori* inclusion and exclusion criteria applied.

PICOST^a^ component	Inclusion criteria	Exclusion criteria
Population	The study examines infectious viral disease in humans during pandemic or epidemic settings.	The study examines bacterial, fungal, parasitic, protozoan, and prion diseases. The study does not explicitly state viral disease has reached epidemic or pandemic status.
Intervention	The study focuses on the contact tracing aspect of NPIs^b^. Contact tracing is measured in terms of the detection of asymptomatic cases and following testing or diagnosis of a confirmed case they may have had close contacts with or random testing. The study may examine single or multiple NPIs, and combinations of contact tracing interventions. Combination contact tracing interventions can also include other interventions such as diagnostic testing, pharmaceutical interventions, and other NPIs.	The study describes NPIs without contact tracing included in the combination intervention.
Comparison	N/A^c^	N/A
Outcomes	The study reports on the following outcomes: Disease incidence: Incidence proportion or attack rate/risk: The percentage of the population that contracts the disease in an at-risk population during a specified time interval. Other included variations will allow cumulative and peak attack rates. Infection rate (or incident rate): An incidence rate is typically used to measure the frequency of occurrence of new cases of infection within a defined population during a specified time frame. Disease transmission: Reproduction number (R0): The basic reproduction number that is used to measure the transmission potential of a disease. Reduction and risk of transmission (primary or secondary) will be abstracted. Mortality: Case fatality proportion: The proportion of deaths within a defined population of interest. Peak excess death rates: A temporary increase in the mortality rate in a given population. Mortality rate: The total number of deaths from a particular cause in one year divided by the number of people alive within the population at mid-year. An example is cumulative death rate. Total deaths: The number of deaths considered all-cause mortality. Hospitalization: This includes both regular and intensive care unit admissions. The study may also report qualitative findings of outcomes from modeling studies.	The study does not report quantitative or qualitative data on the effectiveness of contact tracing.
Settings	No study limits on geography, global findings.	N/A
Study limits	Study type: primary literature (original studies, case studies) or secondary literature (including systematic reviews with the same inclusion criteria) with or without meta-analyses and modeling. The publications are either already printed in peer-reviewed journals, conference proceedings, or in the prepublication print phase.	Study types other than a primary study or a secondary study (ie, commentaries, policy reviews, letters, editorials, and reports).

^a^PICOST: Population, Intervention, Control, Outcomes, Study design and Timeframe.

^b^NPI: nonpharmaceutical intervention.

^c^N/A: not applicable.

## Results

### Study Characteristics

**Figure 1:**
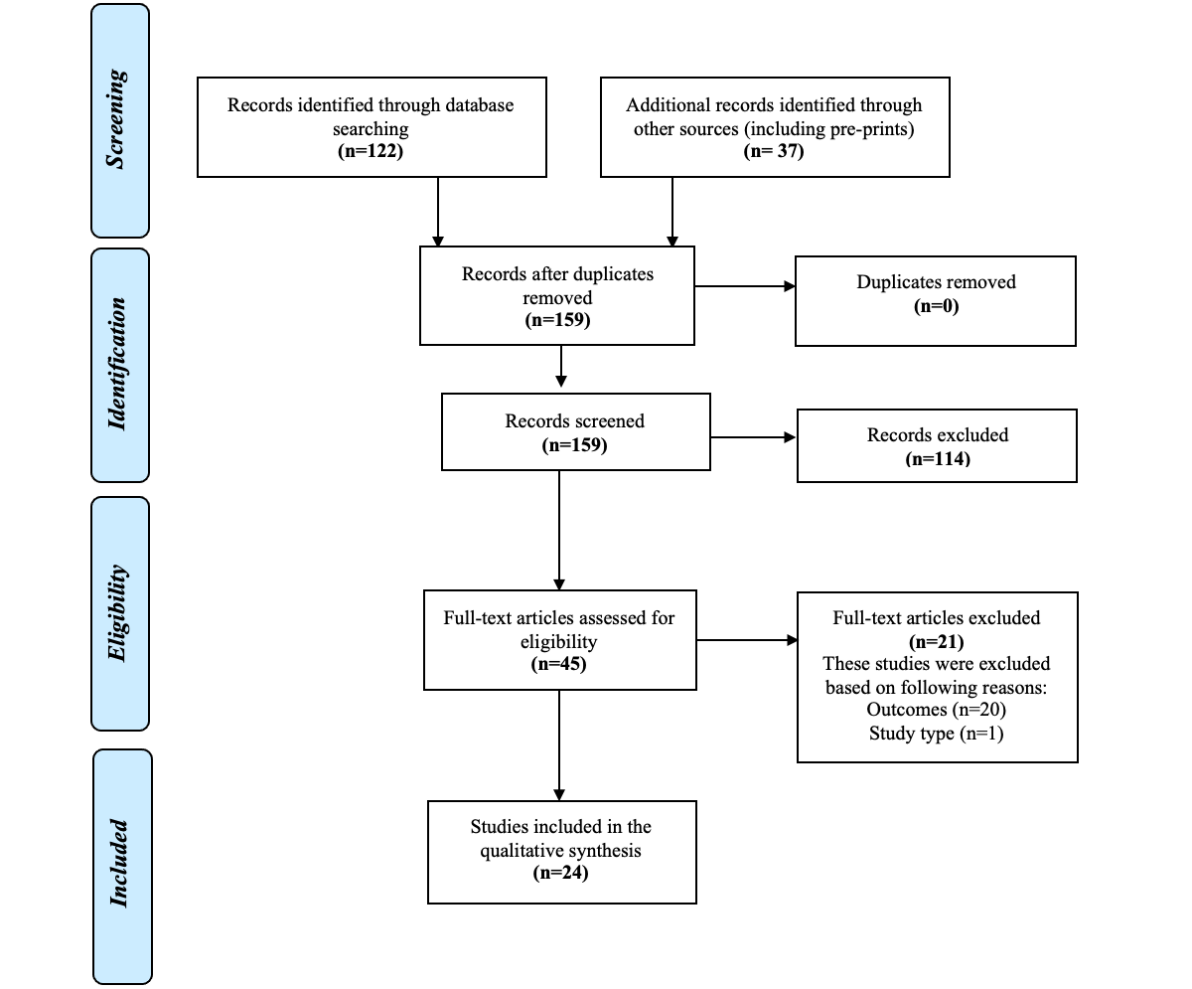
Results of the literature search. Summary of all articles identified by systematic search queries and tracking of articles that were included and excluded across the study screening phases with reasons for exclusion of full texts.

The search strategy yielded a total of 159 unique records, and 45 records (28.3%) were reviewed at the full-text level (Figure 1). A total of 24 (15.1%) studies met the inclusion criteria [13-36], and their characteristics are provided in Table 3 and Table S1 of Multimedia Appendix 1.

Most studies (n=14) [13,16,18,19,21-23,26-28,30,31,34,35] used mathematical modeling, but others were observational studies (n=8) [14,15,20,24,29,32,33,36] and systematic reviews (n=2) [17,25]. These modeling studies used synthetic populations to provide quantitative analyses primarily of COVID-19 evolution to query the effectiveness of contact tracing with other interventions. Identified study settings were global [16,17,25,28,32], nonspecified [19,22,27,31], or included the following countries: Canada [30,35], China [36], India [33], Korea [20,29], Taiwan [14,24], United Kingdom [15,18,21,23], and the United States [13,26,34]. Intervention duration varied across the studies, and both children and adults were targeted. SARS-CoV-2 (n=18) was the most frequently examined viral outbreak [13,14,16-19,21-24,26,27,29-32,34,35], but Nipah virus (n=1) [33] and various influenza A hemagglutinin (H) and neuraminidase (N) subtypes, including H1N1 (n=4) [17,25,28,36] and H7N2 (n=1) [15], were also studied.

**Table 3:** Summary of study characteristics.

Serial number	Study reference	Geography		Study design; level of evidence^a^	Causative virus^b^	Effectiveness outcome(s) assessed
		Location	Continent			
1	Aleta, 2020 [13]	United States	North America	Modeling; 2b	SARS-CoV-2	Hospitalization; incidence
2	Cheng, 2020 [14]	Taiwan	Asia	Observational; 4	SARS-CoV-2	Incidence
3	Eames, 2010 [15]	United Kingdom	Europe	Observational; 4	Influenza A (H7N2)	Incidence; transmission
4	Fiore, 2020 [16]	Global	Global	Modeling; 2b	SARS-CoV-2	Incidence; transmission
5	Fong, 2020 [17]	Global	Global	Systematic review; 2a	Varied (included influenza A subtypes)	Incidence; transmission
6	Goscé, 2020 [18]	United Kingdom	Europe	Modeling; 2b	SARS-CoV-2	Mortality; transmission
7	Hellewell, 2020 [19]	N/R^c^	N/R	Modeling; 2b	SARS-CoV-2	Transmission
8	Jung, 2020 [20]	South Korea	Asia	Observational; 4	SARS-CoV-2	Transmission
9	Keeling, 2020 [21]	United Kingdom	Europe	Modeling; 2b	SARS-CoV-2	Transmission
10	Kretzchmar, 2020 [22]	N/R	N/R	Modeling; 2b	SARS-CoV-2	Transmission
11	Kucharski, 2020 [23]	United Kingdom	Europe	Modeling; 2b	SARS-CoV-2	Transmission
12	Liu, 2020 [24]	Taiwan	Asia	Observational; 4	SARS-CoV-2	Incidence; transmission
13	Mizumoto, 2013 [25]	Global	Global	Systematic review; 4	Influenza A (H1N1)	Transmission
14	Ngonghala, 2020 [26]	United States	North America	Modeling; 2b	SARS-CoV-2	Hospitalization; mortality; transmission
15	Peak, 2020 [27]	N/R	N/R	Modeling; 2b	SARS-CoV-2	Transmission
16	Ross, 2015 [28]	Global	Global	Modeling; 2b	Influenza A (H1N1)	Transmission
17	Son, 2020 [29]	South Korea	Asia	Observational; 4	SARS-CoV-2	Incidence
18	Tang, 2020 [30]	Canada	North America	Modeling; 2b	SARS-CoV-2	Transmission
19	Torneri, 2020 [31]	N/R	N/R	Modeling; 2b	SARS-CoV-2	Transmission
20	Wilasang, 2020 [32]	Global	Global	Observational; 4	SARS-CoV-2	Transmission
21	Wilson, 2020 [33]	India	Asia	Observational; 4	Nipah virus (NiV)	Incidence
22	Worden, 2020 [34]	United States	North America	Modeling; 2b	SARS-CoV-2	Transmission
23	Wu, 2020 [35]	Canada	North America	Modeling; 2b	SARS-CoV-2	Transmission
24	Zhang, 2012 [36]	China	Asia	Observational; 4	Influenza A (H1N1)	Incidence

^a^Adapted from Oxford Levels of Evidence [12]. Level 2a: systematic review with homogeneity of 2b or better studies; level 2b: retrospective cohort, simulation, or modeling studies; level 4: case series or systematic review with heterogeneity of studies.

^b^H#N#: hemagglutinin subtype number and neuraminidase subtype number

^c^N/R: not reported.

### Types of Contact Tracing Interventions and its Combination With Other Interventions

Five studies [15,24,28,36,37] examined contact tracing in a model as a single intervention. Most studies (n=19) [13,14,16-23,25,26,29-35] used a combination of interventions to assess the effectiveness of contact tracing using NPIs with or without PIs. Isolation (ie, separation of diagnosed individuals) and quarantine (ie, restricted movement of presumably infectious individuals) were most frequently combined with contact tracing as a multipronged approach in public health strategies to combat the outbreak. Case detection by diagnostic testing was an additional consideration for the effectiveness of contact tracing. Other interventions with contact tracing included general social distancing NPIs (eg, school closure, mass gathering bans, travel restrictions, workplace policies to limit contact, and nonspecified social distancing to limit public contact), personal protective equipment (eg, mask wearing), increased hygienic practices (eg, handwashing and sanitization procedures), antiviral prophylaxis and/or treatment, symptom monitoring by public health personnel, and screening practices to identify active cases.

Only two studies [22,23] considered digital approaches that included the use of mobile app technology rather than traditional manual contact tracing interventions. No hybrid approaches of traditional and mobile app–based strategies were identified; however, there were comparative analyses between the two strategies.

### Effectiveness of Contact Tracing

Viral disease outcomes associated with the effectiveness of contact tracing were limited to disease incidence, hospitalization, mortality, and transmission (Table 1). The most frequently reported outcomes were transmission (n=19) [13,15-27,30-32,34,35] and incidence (n=9), [13-17,24,28,29,33] whereas hospitalization and mortality outcomes were described in only 3 studies [13,18,26]. All studies reported some degree of effectiveness for contact tracing examined across mild to moderate (R0<1.5) and/or moderate to severe (R0≥1.5) epidemics or pandemics based on the provided R0. Number of contacts traced, timeliness, and compliance with combination intervention implementation, and R0 were found to impact effectiveness. Combination of NPIs with contact tracing was deemed the most effective [26]. One study modeled the effects of contact tracing as a single intervention and it had minimal effects, but the impact was improved by adding social distancing, quarantine, and mask-wearing to case identification. US peak transmission and hospitalizations decreased 10% from the baseline with contact tracing alone and 92%, with a combination of NPIs [26]. Moreover, nationwide mortality changes from baseline improved substantially (US mortality change from baseline: –3% to –64%) by combining contact tracing with other interventions [26]. In a scenario where 50% of symptomatic cases were identified, a 20% effective contact tracing strategy combined with quarantine, isolation, and general social distancing would help reduce the hospital and intensive care unit (ICU) peak daily admissions per 1000 people from 2.35 (95% CI 1.97-2.75) and 1.39 (95% CI, 1.11-1.68) to 0.44 (95% CI 0.28-0.62) and 0.28 (95% CI 0.16-0.42), respectively. With a 40% effective contact tracing strategy, these estimates would further decrease to 0.29 (95% CI 0.18-0.43) and 0.15 (95% CI 0.08-0.26) [13]. Due to intervention heterogeneity and outcome reporting, it was not possible to provide valid head-to-head effectiveness comparisons across studies.

### Transmission

Combinations of NPIs with contact tracing decreased viral transmissibility. Countries that implemented widespread diagnostic SARS-CoV-2 testing with active case detection and prompt isolation were more successful in decreasing the R0 than those that did not use contact tracing (decrease in R0: 0.4-2.2) [32]. A higher decrease in transmission was predicted if contact tracing were combined with case isolation strategies rather than with symptom monitoring [17]. Similarly, transmission would decrease by more than 12% to 64% than with mass testing or self-isolation strategies used alone [23]. Simulations indicated that policies to mitigate SARS-CoV-2 transmission, including contact tracing, isolation, and testing, had similar impacts across geographies [16]. As interventions were added, effectiveness was compounded. Simulations indicated that contact tracing prevented 44% of transmissions from a primary case, and the R0 decreased from 1.85 to 1.13, with shelter in place and public mask-wearing policies coupled with contact tracing, isolation, and quarantine in US COVID-19 settings [34]. Similarly, another modeling study conducted in England observed that SARS-CoV-2 transmission decreased substantially following weekly universal testing, mask-wearing, and contact tracing during a lockdown (effective R0: lockdown lifted and no interventions, 2.56; with interventions, 0.27) [18]. Additionally, when antiviral treatments were added to NPIs, the strategy used resulted in a further decrease in transmission and increased the probability of suppressing the COVID-19 pandemic [31].

Compliance or achievement of combination NPIs with contact tracing and severity of the R0 affected their success. The modeling of isolation measures with contact tracing predicted decreased SARS-CoV-2 transmission (preintervention R0: 1.5; post-intervention R0: 0.5-0.9 based on 20%-100% contact tracing achievement) [19]. Moreover, a higher achievement of contact tracing was required as the R0 increased [19]. One simulation study showed that increased case detection by contact tracing reduced the R0 from 3 to 0.5 when used in combination with other community-enforced personal protection measures such as wearing a mask [35]. Additionally, testing efficacy improved the effectiveness of contact tracing. When both presymptomatic and asymptomatic infections occurred, contact tracing was more effective when combined with testing rather than monitoring, provided the diagnostic test was sensitive enough to detect infections during the incubation period in a COVID-19 model [31]. One modeling study identified many simulated conditions that resulted in SARS-CoV-2 suppression (ie, R0<1) of transmission; the parameters included high (≥60%) contact tracing and testing efficacy to accommodate a range of testing capacities (low to high incidence) [16]. Even with short serial intervals, if social distancing NPIs could decrease R0 to 1.25, then adding active monitoring of about 50% of contacts predicted suppressed transmission (R0<1) [27].

Moreover, the duration and timing of contact tracing interventions influenced their effectiveness on limiting transmission. One modeling study noted that the duration of the combined interventions, including contact tracing, was a necessary consideration for its implementation. Although these measures could abate epidemic-level COVID-19 transmission, it would not prevent resurgence if measures were relaxed or removed [13]. A model with combined isolation and contact tracing predicted that the delay between symptom onset and isolation had the largest role in determining whether a COVID-19 outbreak (R0=1.5) was controllable [19]. Further, early detection of asymptomatic cases with high efficiency of contact tracing and SARS-CoV-2 testing adequately limited the observed transmission in nosocomial settings [20]. In ideal scenarios (assuming no testing and tracing delays and 40% of transmissions occurring before symptom onset), contact tracing could achieve COVID-19 suppression (ie, R0<1; effective R0: social distancing NPIs only, 1.2; add contact tracing to NPIs, 0.8; 95% CI 0.7-1.0). However, if testing delays were greater than 3 days, the most efficient combinatory strategies could not suppress transmission (ie, keep R0<1) in COVID-19 models [22].

Logistical and economic burdens were identified in traditional contact tracing interventions, and alternative means of surveillance and monitoring were considered, including the use of digital contact tracing. Keeling et al [21] computed the distribution of epidemiological, social, and contact tracing characteristics across the population using preliminary estimates of severe COVID-19 transmission. The model predicted that with effective contact tracing, less than 1 in 6 cases will generate any subsequent untraced infections. This approach comes with a high logistical burden, given an average of 36 individuals traced per case [21]. In fact, another US modeling study noted that a 75% improvement in contact tracing resulted in a 10% reduction in nationwide pandemic peak, highlighting its potentially limited ability to scale in a cost-effective manner [26]. Recall bias was a further limitation in contact tracing efforts. One COVID-19 modeling study provided comparative effectiveness to overcome these burdens [22]. Digital contact tracing demonstrated limited superiority over traditional methods in simulation. Mobile app–based tracing was more effective than traditional tracing with limited efficacy (ie, 20% coverage; change in R0: digital, –17.6%; traditional, –2.5%) [22].

### Incidence

Epidemiological studies examining SARS-CoV-2 [14,24,29] or other viruses [12,15,33,36] described the effectiveness of contact tracing on viral disease incidence. Multiple studies provided the effectiveness of contact tracing as part of mitigation and suppression strategies; some reported the number of cases identified [24,36] and contained [24,29,33] through contact tracing, but noted high resource utilization [36]. Four studies provided secondary attack rates following contact tracing [12,14,15,29] and described the temporal differences based on timing of exposure and contact tracing effectiveness [14]. COVID-19 secondary attack rate was higher when the exposure to an index case started within 5 days of symptom onset (1%, 95% CI 0.6%-1.6%) than when the exposure occurred later (0%, 95% CI 0%-4%). Contact tracing also effectively delineated the associated determinants of COVID-19 secondary attack rates. For example, household and nonhousehold family contacts had higher secondary attack rates than those found in health care or other settings (4.6%-8.2% vs 0.1%-0.9%) [14,29], and attack rates were higher among those older than 40 years [14].

Two modeling studies identified that, when used as part of a combination intervention, contact tracing reduced viral disease incidence [16,17]. A systematic review by Fong et al [17] found that contact tracing of influenza A provided modest benefits when infection rates were high, but it was more effective than symptom monitoring when combined with a quarantine strategy. Fiore et al [16] identified the optimal contact tracing capacities when used in combination with isolation, quarantine, and diagnostic testing with variable efficacies (20%-100%) to determine the predicted impact on incidence when compared to the absence of containment strategies.

### Hospitalization and Mortality

The reported effects of contact tracing on COVID-19–related hospitalization and mortality outcomes were limited to three modeling studies [13,18,26]. If contact could be decreased by 40%, then predicted hospitalization and mortality could be reduced by 88% and 64%, respectively, with NPIs including contact tracing [26]. Mortality would be decreased with the addition of contact tracing to suppression and mitigation strategies (ratio of cumulative deaths to no mitigation: 14.5-fold; NPIs with contact tracing: 0.48-fold) [18].

On par with other outcomes, the timing and duration of interventions affected the effectiveness of contact tracing on hospitalization. Compared to no mitigation strategy, substantial reductions in hospitalizations (both normal and ICU admissions) were expected upon the addition of the contact tracing strategy. However, if intervention duration is insufficient (ie, measures are relaxed or removed), then the tracing effort would need to be raised by approximately 50% for hospitals to accommodate the increased number of infections [13].

### Study Quality

Using Oxford Levels of Evidence [12], most studies provided level 2b evidence as modeling summarizations [13,16,18,19,21-23,26-28,30,31,34,35]; 1 systematic review with homogeneous interventions provided level 2a evidence [17]; 1 systematic review provided level 4 evidence due to intervention heterogeneity [25]; and the observational studies provided level 4 evidence [14,15,20,24,29,32,33,36]. Furthermore, 3 studies were preprints [13,16,34] The quality of the evidence identified was moderate to low, as 8 studies were classified as level 4.

## Discussion

### Principal Findings

To the best of our knowledge, this is the first evidence-based review highlighting the impact of contact tracing on the incidence, transmission, hospitalization, and mortality of a viral infectious disease in the context of the COVID-19 pandemic. Contact tracing, in combination with other NPIs or PIs, decreased disease incidence and transmission. A reduction in hospitalizations and mortality of the viral infectious disease was also facilitated by contact tracing. Early, sustained, and layered application of various NPIs, including contact tracing could mitigate and suppress primary outbreaks and prevent more severe secondary or tertiary outbreaks provided that decision-makers consider some important limitations. Retrospective observational and modeling studies suggest the effectiveness of contact tracing and other NPIs are not only largely dependent upon on disease severity and its dynamic R0 values but also on intervention timing, duration, compliance, efficiency, and the number of asymptomatic cases. Thus, an outbreak could be effectively suppressed through strict and early implementation of combined interventions, as long as they can be maintained. The higher the infectivity of the disease and/or the longer the delay in implementation of a measure, the lower would be the resulting effectiveness of the interventions. Additionally, the number of contacts traced and tested without delay and the number of asymptomatic infectious cases are also very important considerations in public health response planning.

It is important to consider these data alongside the limitations of contact tracing—the need for adapting programs based on the local context, resources, and customs; implementation challenges when disease incidence is very high; and limited scalability. To improve scale, the use of digital surveillance tools to track the contacts of people infected with an infectious disease, such as COVID-19, could be key to reducing the number of people infected and reducing the spread of the virus. More countries are implementing digital tools for contact tracing through mobile apps that allow user data to be shared via the device’s GPS and/or Bluetooth capabilities; however, this approach raises concerns about privacy; confidentiality of data; and functional or technological limitations, such as dependency on voluntary adoption, performance-related errors, limited effectiveness in identifying contacts, and restrictions associated with operating systems [38-40]. During our screening, we identified several implementation and theoretical studies regarding the use of digital contact tracing, but most of them lacked outcomes data for inclusion [38,41-49]. A gap remains in understanding the effectiveness of these mobile apps, particularly since limited evidence exists on their effectiveness, although modeling studies have suggested that contact-tracing apps could reduce disease transmission [50]. Notably, there are guidelines set forth by various government agencies to augment traditional contact tracing with digital tools [51].

### Strengths and Limitations

This review has several strengths. To our knowledge, this is a novel review using a rigorous methodology to provide a qualitative synthesis of the evidence related to the effects of contact tracing on viral disease outcomes. Synthesis included studies examining the COVID-19 pandemic caused by SARS-CoV-2 (2019-2020), in addition to historic viral epidemics. This examination provides stakeholders with evidence-based findings to better understand the importance and benefits of timely and strategic implementation of contact tracing in the current social context of a severe pandemic.

Our results should be interpreted in the context of their limitations. From a study design perspective, the search was not comprehensive, as only one database (including its preprint contents) was searched, and no handsearching of included studies or conference proceedings was performed to expediently provide synthesis of the available information. Furthermore, the findings are of limited generalizability due to the relatively small number of identified studies, most of which were of moderate to low quality. Additional considerations need to be made for the large number of modeling studies that were used to derive this transmission-based evidence as opposed to epidemiological findings. Finally, the consideration of contact tracing alone and in combination with various other interventions, as observed in the response to the COVID-19 pandemic, limits the interpretation of the causal role of contact tracing in disease mitigation. However, recent work suggests that comparisons between different permutations of NPIs may still be informative [52].

### Conclusions

This evidence-based review suggests that the proper deployment of strategically layered NPIs that include contact tracing along with other interventions, such as testing, could mitigate and suppress disease burden by decreasing viral disease incidence, transmission, and resulting hospitalizations and mortality. Strict and timely implementation of NPIs is necessary to minimize inefficiencies associated with their limited ability to scale with the surge of outbreaks. Future work should focus on the ability of digital methods to augment traditional contact tracing and its associated privacy and ethical considerations, the accuracy and assumptions of contact tracing models, and the specific effects of vaccines and other PIs on contact tracing.

## Appendix

Multimedia Appendix 1 Supplementary Table 1: abstractions of included studies.

## Abbreviations

ICU: intensive care unit
NPI: nonpharmaceutical intervention
PI: pharmaceutical intervention
R0: reproduction number
SARS: severe acute respiratory syndrome
