# Supplementary material for: Effectiveness of Contact Tracing for Viral Disease Mitigation and Suppression: Evidence-Based Review
Source: JMIR Public Health Surveill. 2021 Oct 6;7(10):e32468. doi: 10.2196/32468 (PMC8496751; doi:10.2196/32468)
Supplement: Multimedia Appendix 1 [file publichealth_v7i10e32468_app1.docx]

**Supplementary Table 1. Study abstractions**

| **Ref**  **No.** | **Ref,**  **Year** | **Infectious Disease** | | **Intervention** | | **Comparison(s)** | **Outcome(s)** | **Result(s)** | **Conclusion(s)** |
| --- | --- | --- | --- | --- | --- | --- | --- | --- | --- |
|  |  | Disease | Year(s) | Type(s) | Duration |  |  |  |  |
| 1 | Aleta, 2020^1^ | COVID-19 | 2020 | Combination interventions  **^a^ contact tracing (with cohort testing);**  [no, 20% and 40%]  ^b^ quarantine;  ^c^ isolation;  ^d^ general social distancing; | Strict lockdown ~8 wks followed by partial lockdown ~4 wks | Intervention vs. no intervention; combination of interventions | Hospitalization; incidence | Hospitalization  *Peak daily hospitalization/1000 (95% CI)*  No intervention:  Hospital, 4.57 (4.10–5.03);  ICU, 2.56 (2.21–2.91)  ^a,b,c,d^ interventions, detection 30%:  No tracing -  Hospital 2.70 (2.29–3.12),  ICU 1.58 (1.27–1.88);  Tracing 20% -  Hospital 0.86 (0.65–1.10),  ICU 0.55 (0.39–0.72);  Tracing 60% -  Hospital 0.35 (0.21–0.50),  ICU 0.22 (0.12–0.34);  ^a,b,c,d^ interventions, detection 50%:  No tracing -  Hospital 2.35 (1.97–2.75),  ICU 1.39 (1.11–1.68);  Tracing 20% -  Hospital 0.44 (0.28–0.62),  ICU 0.28 (0.16–0.42);  Tracing 40% -  Hospital 0.29 (0.18–0.43),  ICU 0.15 (0.08–0.26);  Incidence  *Peak daily infection incidence/1,000 people (95% CI)*  No intervention, 25.2 (23.8-26.4);  ^a,b,c^ interventions, ~1.5 (1–2)  [Note: R0 = 2.5] | Gradually decreasing social distancing interventions, when combined with testing, contact tracing, isolation, and quarantine presents a viable course for managing and mitigating COVID-19 spread. |
| 2 | Cheng, 2020^2^ | COVID-19 | 2020 | Combination interventions  **^a^ contact tracing (with cohort testing);**  ^b^ quarantine | Quarantined for 14 days after last exposure | None | Incidence | Incidence  *Secondary attack rate* (95% CI)  ^a,b^ interventions, exposure ≤ 5 days of symptom onset, 1.0% (95% CI, 0.6%–1.6%);  ^a,b^ interventions, exposure > 5 days of symptom onset, 0% (0%–0.4%);  ^a,b^ interventions, exposure before symptoms, 0.7% (0.2%–2.4%);  ^a,b^ interventions, household exposure, 4.6% (2.3%–9.3%);  ^a,b^ interventions, non-household exposure, 5.3% (2.1%–12.8%).  ^a,b^ interventions, age subgroup analysis:  40 to 59 years, 1.1% (0.6%–2.1%);  ≥ 60 years, 0.9% (0.3%–2.1)  [Note: R0, N/P] | COVID-19 has high secondary attack rates preceding and immediately following symptom onset. Contact tracing and quarantine are most likely to be effective when targeting these infection scenarios. |
| 3 | Eames, 2010^3^ | Influenza A (H7N2) | 2007 | Single intervention  **^a^ contact tracing (with cohort testing);**  Combination interventions  **^a^ contact tracing (with cohort testing);**  ^f^ antiviral prophylaxis | N/P | None | Incidence; transmission | Incidence  *Estimated secondary cases (in the absence of treatment)*  ^a^ intervention, range 0.1–2.7  Transmission  *Probability of transmission*  ^a,f^ interventions, home, 0.11%;  ^a,f^  interventions, work, 0.095%;  ^a,f^  interventions, other contact, 0.0055%  [Note: R0, N/P] | Rapid treatment  or prophylaxis combined with contact tracing is expected to reduce transmission probability. Home contact is most likely to transmit disease, followed by work, and then in other settings. |
| 4 | Fiore, 2020^4^ | COVID-19 | 2020 | Combination interventions  **^a^ contact tracing (with cohort testing);**  ^c^ isolation | N/P | None | Incidence; transmission | Incidence  *Daily incidence growth using tests per thousand (proxy)*  ^a,c^  interventions, growth rate 15% and % contacts traced:  20%, 0.7;  40%, 0.7;  60%, 0.7;  80%, 0.7;  100%, 0.7;  ^a,c^  interventions, growth rate 25% and % contacts traced:  20%, 3;  40%, 2;  60%, 1.7;  80%, 1.7;  100%; 1.7;  ^a,c^  interventions, growth rate 35% and % contacts traced:  20%, 30;  40%, 15;  60%, 4.5;  80%, 3.6;  100%, 3.6;  Transmission  *Effective R0*  ^a,c^ interventions, R0< 0 at 15-35% growth rates when contact tracing ≥60% and testing capacity between 0.7 and 3.6 per thousand;  ^a,c^ interventions, R0 ~ 1 at 15-35% growth rates when contact tracing ≤60% and testing capacity between 0.7 and 4.5 per thousand;  ^a,c^ interventions, R0 > 1 at 25-35% growth rates when contact tracing ≤40% and testing capacity between 0.7 and 30 per thousand.  [Note: R0 or R*_effective_* ~1] | Contact tracing efficacy and testing capacity are interconnected in their ability to help contain transmission. Contact tracing efficacy of ≥ 60% enabled containment of transmission at growth rates of 15-35%. |
| 5 | Fong, 2020^5^  [Note: systematic review] | Influenza A (H1N1, H5N1) | Varied (2003 – 2009) | Combination interventions  **^a^ contact tracing;**  ^b^ quarantine;  ^c^ isolation;  ^f^ antiviral prophylaxis and treatment;  ^g^ symptom monitoring;  ^h^ hygiene / disinfection practices | Varied | No intervention vs intervention; combination of interventions; timing of interventions | Incidence; transmission | Incidence  *Attack rate*  If R0=1.8,  No intervention, 74%;  ^b,c,f^  interventions at 50% compliance, 40%;  ^a,b,c,f^ interventions at 50% compliance, 34%.  Transmission  *Effective R0*  If R0=1.54,  ^a,b^ interventions were more effective to lower transmission than ^a,g^ interventions with shorter delays in contact tracing and a larger fraction of contacts traced;  diseases like influenza A with short incubation periods were strongly influenced by contact tracing delays.  *Timing of peak*  If R0=1.58,  No intervention, 10.3-wks peak time;  ^a,b,c,f,h^ interventions with 30% case detection rate, 7.6-wks peak time;  [Note: R0 range, 1.34 – 21] | Effectiveness of these combination of interventions is affected by the R0 and the case detection rate.  With 90% isolation, it is insufficient to guarantee control, but effective contact tracing together with isolation of symptomatic cases is predicted to readily control outbreaks with high R0. |
| 6 | Goscé, 2020^6^ | COVID-19 | 2020 | Combination interventions  **^a^ contact tracing (with universal testing);**  ^b^ quarantine;  ^c^ isolation;  ^d^ general social distancing (prolonged lockdown);  ^e^ mask-wearing | ~3 weeks | No intervention vs intervention | Mortality; transmission | Mortality  *Ratio of cumulative deaths compared to prolonged lockdown*  No intervention, 14.5;  ^a,b,c,d,e^ interventions, 0.48.  Transmission  *Effective R0*  No intervention, 2.56  ^a,b,c,d,e^ interventions, 0.27  [Note: R0=2.56] | To achieve suppression, a combination of weekly universal testing, contact tracing, and use of facemasks during lockdown are needed.  This reduces deaths by 48% compared with lockdown alone but requires high uptake and sustained local effort. |
| 7 | Hellewell, 2020^7^ | COVID-19; SARS-coronavirus | 2020; 2003 respectively | Combination interventions  **^a^ contact tracing**  **with 20-100% achievement**;  ^c^ isolation | 3 months | Intervention vs no intervention; timing of interventions | Transmission | Transmission  *Effective R0*    No intervention, 1.5;  ^c^ intervention, 1.25;  ^a,c^ interventions at % levels of achievement -  20%, 0.9;  40%, 0.8;  60%, 0.7;  80%, 0.6;  100%, 0.5.  Effective R0 –  No intervention, 2.5;  ^c^ intervention, 2.1;  ^a,c^ interventions at % levels of achievement -  20%, 1.8;  40%, 1.6;  60%, 1.2;  80%, 0.8;  100%, 0.6.  [Note: R0 range, 1.5 – 3.5] | Isolation and contact tracing decreased transmission, but if R0 are high then outbreak containment will require very high levels achievement in the intervention.  For example with 90% control of outbreaks, 80% of contacts need to be traced and isolated if R0=2.5 whereas only 20% need to be traced and isolated when R0=1.25  Majority of scenarios with an R0 of 1.5 were controllable with less than 50% of contacts successfully traced. For R0 of 2.5, more than 70% of contacts had to be traced. For an R0 of 3.5 more than 90% of contacts had to be traced. The delay between symptom onset and isolation had the largest role in determining whether an outbreak was controllable when R0 was 1.5. |
| 8 | Jung, 2020^8^ | COVID-19 | 2020 | Combination interventions  **^a^ contact tracing (with cohort testing);**  ^c^ isolation | 3 weeks | None | Transmission | Transmission  *Number of secondary cases*  ^a,c^ interventions, 3 positive cases traced resulted in 1,206 contacts, 96% of whom were tested and 18 were positive.  [Note: R0 N/P] | Rapid detection of asymptomatic cases through contact tracing and testing effectively contained a potential nosocomial COVID-19 outbreak. |
| 9 | Keeling, 2020^9^ | COVID-19 | 2020 | Combination interventions  **^a^ contact tracing;**  ^c^ isolation | N/P | None | Transmission | Transmission  *Effective R0*  ^a,c^ interventions, 71% of contacts need to be traced to contain transmission (R0 < 1).  With 100% of close contacts traced before becoming infectious, R0 = 0.18.  [Note: R0=3] | The key to contact tracing success is  rapid detection and isolation of the majority of a case’s contacts. However, due to the uncertainties inherent in contact tracing, some untraceable and unidentifiable contacts are expected, requiring additional mitigation strategies. |
| 10 | Kretzchmar, 2020^10^ | COVID-19 | 2020 | Single interventions  ^c^ isolation;  ^d^ general social distancing  Combination interventions  **^a^ contact tracing (with cohort testing);**  **^a^_D_ digital contact tracing;**  ^c^ isolation;  ^d^ general social distancing  with  80-100% coverage and  0, 3- and 7- days testing delay | Up to 7 days | Combination of interventions; timing of interventions | Transmission | Transmission  *Effective R0 (95% CI)*  At 100% coverage,  ^d^ intervention, 1.2;  ^c^ intervention, 1.0 (0.9–1.1);  ^a,c,d^ interventions, 0.8 (0.7–0.9);  At 80% coverage,  ^a,c,d^ interventions, 0.8 (0.7–1.0).  ^a^ intervention, >1 (testing delay exceeds 0 days);  ^a^_D_ intervention, <1 (with even a delay of up to 2 days).  *Reduced R0*  With testing delays,  ^a,c,d^ interventions, 0-day testing delay, 79.9%;  ^a,c,d^ interventions, 3-days testing delay, 41.8%;  ^a,c,d^  interventions, 7-days testing delay, 4.9%.  [Note: R0=1] | In the current situation of de-escalation of physical distancing, contact tracing could be critical to continue the containment.  Reduction in transmission is largely dependent upon minimizing testing delays.  App-based tracing alone remains more effective  than conventional tracing since conventional contact tracing has  a longer tracing delay and lower tracing coverage  app based technology.  Less than 3 days between symptom onset and testing and isolation of an index is essential for successful contact tracing. |
| 11 | Kucharski, 2020^11^ | COVID-19 | 2020 | Combination interventions  **^a^ contact tracing;**  **^a^_D_ digital contact tracing;**  ^b^ quarantine; ^c^ isolation;  ^d^ general social distancing (limit to 4 daily contacts) | N/P | No intervention; combinations of interventions | Transmission | Transmission  *Cases with R0>1*  ^a,b,c^ interventions, 27% with work or school contact tracing;  ^a,b,c^ interventions, 26% with contract tracing of acquaintances;  ^a,b,c^  interventions, 21% with contact tracing of all contacts;  ^a^_D_^,b,c^ interventions, 30% with app-based contact tracing;  ^a, a^_D_^,b,c^ interventions, 23% with manual and app-based contact tracing;  ^a,b,c,d^ interventions, 21% with manual contact tracing;  ^a,a^_D_^,b,c,d^ interventions, 20% with manual and app-based contact tracing  *Mean reduction in effective R0*  (from baseline = no interventions)  ^a,b,c^ interventions, 53% with work or school contact tracing;  ^a,b,c^ interventions, 57% with contract tracing of acquaintances;  ^a,b,c^ interventions, 64% with contact tracing of all contacts;  ^a^_D_^,b,c^ interventions, 47% with app-based contact tracing;  ^a,a^_D_^,b,c^ interventions_,_ 61% with manual and app-based contact tracing;  ^a,b,c,d^ interventions, 64% with manual contact tracing;  ^a, a^_D_^,b,c,d^ interventions, 66% with manual and app-based contact tracing.  [Note: R0=2.6] | Effectiveness of manual contact tracing, social distancing, and the proportion of asymptomatic patients most influenced disease transmission. For manual contact tracing to be effective, hundreds of thousands of patients need to be contacted and quarantined, presenting formidable logistical challenges. Digital contact tracing using app-based technology provided an additive, but limited reduction in transmission. |
| 12 | Liu, 2020^12^ | COVID-19 | 2020 | Single intervention  **^a^ contact tracing;**  ^b^ quarantine; ^i^ screening (airport) | 76 days | None | Incidence; transmission | Incidence  *n (% total / % asymptomatic on arrival)*  ^a^ intervention, 52 (16.2 / 28.3)  ^b^ intervention, 89 (27.7 / 39.6)  ^i^ intervention, 105 (32.7 / 0)  Transmission  *R0 (95% CI)*  ^a^ intervention, 0.15 (0.00–0.30)  ^b^ intervention, 0.04 (0.00–0.09)  ^i^ intervention, 0 (0–0)  [Note: R0=1.2] | Contact tracing was the least effective of the identification routes evaluated in this study. However, contact tracing was able to identify cases who never developed symptoms, indicating its importance for disrupting community transmission. |
| 13 | Mizumoto, 2013^13^  [Note: systematic review] | Influenza A (H1N1) | 2009-2011 | Combination interventions  **^a^ contact tracing;**  ^f^ antiviral prophylaxis | Varied | Combination of interventions; timing of interventions | Transmission | Transmission  *Pooled secondary infection risk across 17 studies (median)*  ^a^ intervention, 16.6%;  ^a,f^ interventions, 2.1%  [Note: R0, N/P] | Antiviral prophylaxis coupled with contact tracing lowered secondary infection risk, but authors were unable to estimate the effectiveness in reducing transmission due to high heterogeneity in the model. |
| 14 | Ngonghala, 2020^14^ | COVID-19 | 2020 | Single intervention  ^d^ general social distancing (40% reduction from baseline);  Combination interventions  **^a^ contact tracing;**  ^b^ quarantine;  ^c^ isolation;  ^d^ general social distancing (40% reduction from baseline);  ^e^ mask-wearing (>70% efficacy) | Varied | Combination of interventions; timing of interventions | Hospitalization; mortality; transmission; | Hospitalization  *Decrease from no intervention*  ^a,b,c,d,e^ interventions, ^~^25% in NY and US with 10% reduction in contact rate;  ^a,b,c,d,e^  interventions, ^~^50% in NY and US with 20% reduction in contact rate;  ^a,b,c,d,e^  interventions, 77% in NY with 30% reduction in contact rate;  ^a,b,c,d,e^ interventions, 73% in US with 30% reduction in contact rate;  ^a,b,c,d,e^  interventions, 92% in NY with 40% reduction in contact rate;  ^a,b,c,d,e^  interventions, 88% in US with 40% reduction in contact rate;  Transmission  *Decrease in number of new peak cases*  ^a^ intervention at baseline, 27% in NY;  ^a^ intervention at baseline, 22% in US;  ^a^ intervention with 75% improvement, 37% in NY;  ^a^ intervention with 75% improvement, 36% in US;  Mortality  *Cumulative decrease from baseline*  ^a,b,c,d^ interventions, 13% in NY, ending April 2020;  ^a,b,c,d^ interventions, 28% in US, ending May 2020;  ^a,b,c,d,e^  interventions, 13% in NY, ending May 2020;  ^a,b,c,d,e^  interventions, 28% in US, ending May 2020;  ^a,b,c,d,e^  interventions, 68% in NY, ending June 2020;  ^a,b,c,d,e^  interventions, 76% in NY, duration March 1 – December 2020;  ^a,b,c,d,e^  interventions, 63% in NY, duration January 20 – December 2020;  ^a,b,c,d,e^  interventions, 48% in NY, duration March 22 – December 2020;  ^a,b,c,d,e^  interventions, 53% in NY, duration January 20 – December 2020;  [Note: R0=2.07] | Timing and duration of interventions greatly influenced effectiveness of social distancing (including contact tracing) on COVID-19outcomes in a major metropolitan city. Authors noted that investing resources to achieve contact tracing expectations beyond baseline may not be cost effective. |
| 15 | Peak, 2020^15^ | COVID-19 | 2020 | Single interventions  **^a^ contact tracing;**  ^b^ quarantine;  Combination interventions  ^b^ quarantine  ^d^ social distancing  or  ^a^ contact tracing  ^d^ social distancing  social | N/A | No intervention vs intervention; combination of interventions | Transmission | Transmission  *Effective R0 (95% CI)*  High feasibility scenario, serial interval 4.8 days:  ^a^ intervention, 1.55 (0.65–2.7);  ^b^ intervention, 0.57 (0.32–1.05);  High feasibility scenario, serial interval 7.5 days:  ^a^ intervention, 0.54 (0.32–0.98);  ^b^ intervention, 0.49 (0.34–0.97);  *Median reduction in Effective R0*  High feasibility scenario, serial interval 4.8 days:  ^a^ intervention, 3%;  ^b^ intervention, 17%;  High feasibility scenario, serial interval 4.8 days, R0 1.25 (social distancing):  ^a^ intervention,  3.2% if 10% contacts traced,  15% if 50% of contacts traced,  33% if 90% of contacts traced;  ^b^ intervention,  5.8% if 10% contacts traced,  32% if 50% of contacts traced,  66% if 90% of contacts traced.  [Note: R0=2.2] | The effectiveness of quarantine vs. active monitoring on controlling transmission depends on efficacy, disease serial interval, and the extent of pre-symptomatic transmission. Each intervention faces scalability challenges in real-world circumstances, but both interventions have potentially synergistic effects with other scalable interventions such as social distancing. |
| 16 | Ross, 2015^16^ | Influenza A (H1N1) | 2009 | Single Intervention  **^a^ contact tracing**  Combination interventions  ^a^ contact tracing;  ^f^ antiviral prophylaxis | N/P | No intervention vs intervention | Transmission | Transmission  *Median probability of a major outbreak*  No intervention, 0.67;  ^f^ intervention, 0.43;  ^a 0.1–0.5 efficiency^ intervention, 0.36;  ^a 0.1–0.5 efficiency,f^ interventions, 0.33  [Note: R0, N/P] | Contact tracing reduced the probability of a major H1N1 outbreak over no intervention or use of antivirals alone with tracing efficacy being the primary determinant of effectiveness. The benefit from antiviral intervention was considerable, but dependent on timing of treatment and not found to be synergistic with contact tracing. |
| 17 | Son, 2020^17^ | COVID-19 | 2020 | Combination interventions  **^a^ contact tracing;**  ^b^ quarantine | N/P | None | Incidence | Incidence  *Household secondary attack rate (95% CI)*  ^a,b^ interventions, 8.2% (4.7–12.9)  [Note: R0, N/P] | Contact tracing and quarantine can control outbreaks of limited scope but are unlikely to provide long-term control unless combined with sustainable containment strategies. |
| 18 | Tang, 2020^18^ | COVID-19 | 2020 | Combination interventions  **^a^ contact tracing;**  ^b^ quarantine;  ^c^ isolation;  ^d^ general social distancing;  ^e^ mask-wearing (and other PPE) | N/P | Combination of interventions | Transmission | Transmission  *Effective R0 (max/min if 7.8 days for diagnosis, max/min if 0.18 transmission probability)*  10–40% contact tracing efficacy  ^a,b,c,e^ interventions,  3.3/2.3, 3.0/2.7 at assumed contact rate;  Limited social distancing -  ^a,b,c,d,e^ interventions,  2.4/1.7, 2.8/1.4 at 25% reduced contact rate;  ^a,b,c,d,e^ interventions,  1.6/1.3, 1.2/1.0 at 50% reduced contact rate;  ^a,b,c,d,e^ interventions,  0.8/0.5, 0.8/0.5 at 75% reduced contact rate;  Moderate social distancing -  ^a,b,c,d,e^ interventions,  2.8/2.0, 2.9/2.0 at assumed contact rate;  ^a,b,c,d,e^ interventions,  2.1/1.5, 2.0/1.2 at 25% reduced contact rate;  ^a,b,c,d,e^ interventions,  1.4/1.0, 1.1/0.9 at 50% reduced contact rate;  ^a,b,c,d,e^ interventions,  0.7/0.5, 0.6/0.5 at 75% reduced contact rate;  Severe social distancing -  ^a,b,c,d,e^ interventions,  2.3/1.6, 2.0/1.2 at assumed contact rate;  ^a,b,c,d,e^ interventions,  1.7/1.3, 1.2/1.0 at 25% reduced contact rate;  ^a,b,c,d,e^ interventions,  1.2/0.8, 1.0/0.8 at 50% reduced contact rate;  ^a,b,c,d,e^ interventions,  0.59/0.4, 0.5/0.4 at 75% reduced contact rate.  [Note: R0, range 0.4-3.3] | The efficacy of contact rate reduction and combination of interventions including contact tracing, quarantine, and isolation are more impactful than severe social distancing measures at controlling COVID-19 spread. At least 50-60% contact tracing efficacy will be required to control transmission under the conditions assumed. |
| 19 | Torneri, 2020^19^ | COVID-19 | 2020 | Combination interventions  **^a^ contact tracing [with cohort testing];**  ^b^ quarantine;  ^c^ isolation;  ^f^ antiviral prophylaxis; | N/P | Combination of interventions | Incidence | Incidence  ^a^ intervention 25% effective, ~58%;  ^a^ intervention 50% effective, ~55%;  ^a^ intervention 75% effective, ~53%;  ^a^ 25% effective^,b,c^ interventions, ~50%;  ^a^ 50% effective^,b,c^ interventions, ~42%;  ^a^ 75% effective^,b,c^ interventions, ~40%;  ^a^ 25% effective^,b,c,f^ interventions, ~36%;  ^a^ 50% effective^,b,c,f^ interventions, ~22%;  ^a^ 75% effective^,b,c,f^ interventions, 18%;  [Note: R0=2.5 for symptomatic patients and R0=1.375 for asymptomatic patients] | The benefit of increased contact tracing effectiveness increases considerably with each additional intervention it is combined with (testing, antivirals upon diagnosis, or all combined). |
| 20 | Wilasang, 2020^20^ | COVID-19 | 2020 | Single intervention  ^d^ general social distancing (including lockdown)  Combination interventions  **^a^ contact tracing [with cohort testing];**  ^b^ quarantine;  ^c^ isolation | Varied | Combination of interventions | Transmission | Transmission  *Decreased R0*  ^a,b,c^ interventions > ^d^ interventions at reducing R0  [Note: Delta R0, range 0.4-2.2] | Countries that implemented liberal testing with active case finding and prompt isolation, combined with contact tracing and quarantine, were more successful in reducing transmission than countries that relied on exclusive social distancing measures including lockdown. |
| 21 | Wilson, 2020^21^ | Nipah | 2018-2019 | Combination interventions  **^a^ contact tracing;**  ^b^ quarantine;  ^c^ isolation | N/P | None | Incidence | Incidence  *Secondary attack rate*  ^a,b,c^ interventions, 0% | Effective contact tracing can limit secondary attacks; strongly recommended public policy for individuals to crate daily social contact diaries in the time of COVID-19. |
| 22 | Worden, 2020^22^ | COVID-19 | 2020 | Single intervention  ^e^ mask-wearing  Combination interventions  **^a^ contact tracing;**  ^d^ general social distancing;  ^e^ mask-wearing | ~90 days | Combination of interventions | Transmission | Transmission  *Decreased effective R0*  ^e^ interventions, 8.6%  ^d,e^ interventions, 35.4%  ^a,e^ interventions, 44%  [Note: R0, range 0.3-3.0] | Contact tracing combined with wearing cloth masks decreased transmission of COVID-19 in a large metropolitan US city. |
| 23 | Wu, 2020^23^ | COVID-19 | 2020 | Combination interventions  **^a^ contact tracing;**  ^d^ general social distancing;  ^e^ mask-wearing | ~28 to 45 days | Intervention vs no intervention | Transmission | Transmission  *Effective R0*  No intervention, 3  ^a,d,e^ interventions, 0.5  Time-dependent change in effective R0 with extended duration of interventions.  [Note: R0, range 0.5-3.0] | Improved case detection by contact tracing lowered transmission, but the role of community enforced personal protection measures are essential to support strict social distancing measures to effectively lower R0. |
| 24 | Zhang, 2012^24^ | Influenza A (H1N1) | 2009 | Single interventions  **^a^ contact tracing;**  ^b^ quarantine;  ^d^ general social distancing (border restrictions);  ^g^ symptom monitoring (travelers);  ^i^ screening (border, hospital) | ~4.5 months | None | Incidence | Incidence  *Case detection*  ^a,b^ interventions, 18.7%;  ^d,i^ interventions, 21.8%;  ^g,i^ interventions, 59.4%;    [Note: Model R0 = 2.2] | Various strategies to detect influenza A cases were effective, but resource utilization was high to achieve success. |

**References**

1. Aleta A, Martin-Corral D, Pastore YPA, et al. Modeling the impact of social distancing, testing, contact tracing and household quarantine on second-wave scenarios of the COVID-19 epidemic. *medRxiv.* 2020.

2. Cheng HY, Jian SW, Liu DP, Ng TC, Huang WT, Lin HH. Contact Tracing Assessment of COVID-19 Transmission Dynamics in Taiwan and Risk at Different Exposure Periods Before and After Symptom Onset. *JAMA Intern Med.* 2020.

3. Eames KT, Webb C, Thomas K, Smith J, Salmon R, Temple JM. Assessing the role of contact tracing in a suspected H7N2 influenza A outbreak in humans in Wales. *BMC Infect Dis.* 2010;10:141.

4. Fiore VG, DeFelice N, Glicksberg BS, et al. Containment of future waves of COVID-19: simulating the impact of different policies and testing capacities for contact tracing, testing, and isolation. *medRxiv.* 2020.

5. Fong MW, Gao H, Wong JY, et al. Nonpharmaceutical Measures for Pandemic Influenza in Nonhealthcare Settings-Social Distancing Measures. *Emerg Infect Dis.* 2020;26(5):976-984.

6. Goscé L, Phillips PA, Spinola P, Gupta DRK, Abubakar PI. Modelling SARS-COV2 Spread in London: Approaches to Lift the Lockdown. *J Infect.* 2020.

7. Hellewell J, Abbott S, Gimma A, et al. Feasibility of controlling COVID-19 outbreaks by isolation of cases and contacts. *Lancet Glob Health.* 2020;8(4):e488-e496.

8. Jung J, Hong MJ, Kim EO, Lee J, Kim MN, Kim SH. Investigation of a nosocomial outbreak of coronavirus disease 2019 in a paediatric ward in South Korea: successful control by early detection and extensive contact tracing with testing. *Clin Microbiol Infect.* 2020.

9. Keeling MJ, Hollingsworth TD, Read JM. Efficacy of contact tracing for the containment of the 2019 novel coronavirus (COVID-19). *J Epidemiol Community Health.* 2020.

10. Kretzschmar ME, Rozhnova G, Bootsma MCJ, van Boven M, van de Wijgert J, Bonten MJM. Impact of delays on effectiveness of contact tracing strategies for COVID-19: a modelling study. *Lancet Public Health.* 2020.

11. Kucharski AJ, Klepac P, Conlan AJK, et al. Effectiveness of isolation, testing, contact tracing, and physical distancing on reducing transmission of SARS-CoV-2 in different settings: a mathematical modelling study. *Lancet Infect Dis.* 2020.

12. Liu JY, Chen TJ, Hwang SJ. Analysis of Imported Cases of COVID-19 in Taiwan: A Nationwide Study. *Int J Environ Res Public Health.* 2020;17(9).

13. Mizumoto K, Nishiura H, Yamamoto T. Effectiveness of antiviral prophylaxis coupled with contact tracing in reducing the transmission of the influenza A (H1N1-2009): a systematic review. *Theor Biol Med Model.* 2013;10:4.

14. Ngonghala CN, Iboi E, Eikenberry S, et al. Mathematical assessment of the impact of non-pharmaceutical interventions on curtailing the 2019 novel Coronavirus. *Math Biosci.* 2020;325:108364.

15. Peak CM, Kahn R, Grad YH, et al. Individual quarantine versus active monitoring of contacts for the mitigation of COVID-19: a modelling study. *Lancet Infect Dis.* 2020.

16. Ross JV, Black AJ. Contact tracing and antiviral prophylaxis in the early stages of a pandemic: the probability of a major outbreak. *Math Med Biol.* 2015;32(3):331-343.

17. Son H, Lee H, Lee M, et al. Epidemiological characteristics of and containment measures for COVID-19 in Busan, Korea. *Epidemiol Health.* 2020;42:e2020035.

18. Tang B, Scarabel F, Bragazzi NL, et al. De-Escalation by Reversing the Escalation with a Stronger Synergistic Package of Contact Tracing, Quarantine, Isolation and Personal Protection: Feasibility of Preventing a COVID-19 Rebound in Ontario, Canada, as a Case Study. *Biology (Basel).* 2020;9(5).

19. Torneri A, Libin P, Vanderlocht J, Vandamme AM, Neyts J, Hens N. A prospect on the use of antiviral drugs to control local outbreaks of COVID-19. *BMC Med.* 2020;18(1):191.

20. Wilasang C, Sararat C, Jitsuk NC, et al. Reduction in effective reproduction number of COVID-19 is higher in countries employing active case detection with prompt isolation. *J Travel Med.* 2020.

21. Wilson A, Warrier A, Rathish B. Contact tracing: a lesson from the Nipah virus in the time of COVID-19. *Trop Doct.* 2020:49475520928217.

22. Worden L, Wannier R, Blumberg S, Ge AY, Rutherford GW, Porco TC. Estimation of effects of contact tracing and mask adoption on COVID-19 transmission in San Francisco: a modeling study. *medRxiv.* 2020.

23. Wu J, Tang B, Bragazzi NL, Nah K, McCarthy Z. Quantifying the role of social distancing, personal protection and case detection in mitigating COVID-19 outbreak in Ontario, Canada. *J Math Ind.* 2020;10(1):15.

24. Zhang Y, Yang P, Liyanage S, et al. The characteristics of imported cases and the effectiveness of outbreak control strategies of pandemic influenza A (H1N1) in China. *Asia Pac J Public Health.* 2012;24(6):932-939.
